# Associations Between Concentrations of Vitamin D3, Vitamin B12, and Folate and the Well-Being of Medical Students

**DOI:** 10.3390/nu18101559

**Published:** 2026-05-14

**Authors:** Beata Cieślikiewicz, Anna Bieńkowska, Stanisław Maksymowicz, Justyna Dorf, Katarzyna Młynarska-Antochów, Patrycja Wiącek, Marzena Kułakowska-Foks, Joanna Chomiczewska, Gracjan Szczubełek, Robert Świerszcz, Łukasz Dąbrowski, Blanka Wolszczak-Biedrzycka

**Affiliations:** 1 Department of Psychology and Sociology of Health and Public Health, University of Warmia and Mazury in Olsztyn, 10-082 Olsztyn, Poland; beata.cieslikiewicz@uwm.edu.pl (B.C.); anna.bienkowska@uwm.edu.pl (A.B.); stanislaw.maksymowicz@uwm.edu.pl (S.M.); 2 Department of Clinical Laboratory Diagnostics, Medical University of Białystok, 15-269 Białystok, Poland; justyna.dorf@umb.edu.pl; 3 Department of Nursing, University of Warmia and Mazury in Olsztyn, 10-082 Olsztyn, Poland; katarzyna.mlynarska@uwm.edu.pl; 4 Provincial Psychiatric Hospital, 10-228 Olsztyn, Poland; chmielewskapatrycja20001@gmail.com (P.W.); fox-1103@qp.pl (M.K.-F.); j-chomiczewska@wp.pl (J.C.); 5 Emergency Department, Regional Specialized Children’s Hospital in Olsztyn, 10-561 Olsztyn, Poland; g.szczubelek980w@gmail.com (G.S.); robert15120@gmail.com (R.Ś.); 6 Diagnostyka Medical Laboratory, 10-692 Olsztyn, Poland; lukaszdabrowski.com@gmail.com

**Keywords:** vitamin D3, vitamin B12, folate, well-being

## Abstract

**Introduction:** Medical students are particularly susceptible to nutritional deficiencies and mental health problems due to intensive study demands, stress, and lifestyle factors. Vitamin D3, vitamin B12, and folate deficiencies have been implicated in mental well-being, although evidence remains inconsistent. **Objective:** To assess the prevalence of vitamin D3, vitamin B12, and folate deficiencies among medical students at the University of Warmia and Mazury in Olsztyn, and to explore associations between serum concentrations of these vitamins, lifestyle factors, and self-reported well-being. **Materials and Methods:** The study included 97 medical students. Serum vitamin concentrations were measured using electrochemiluminescence immunoassay. Well-being was assessed with the WHO-5 Well-Being Index. Group comparisons were performed using non-parametric tests, and a Poisson regression model was applied as an exploratory analysis to examine associations between selected lifestyle factors and well-being. **Results:** Vitamin D3 deficiency was observed in 78% of students, folate deficiency in 20%, and vitamin B12 deficiency in 8%. In unadjusted analyses, differences in serum vitamin D3 and vitamin B12 concentrations were observed between students with lower and higher self-reported well-being, whereas folate concentrations did not differ. However, after correction for multiple testing using the Benjamini–Hochberg procedure, none of these associations remained statistically significant. Exploratory regression analysis suggested that physical activity and gender may be associated with well-being, while no association with vitamin D3 supplementation was observed. **Conclusions:** Vitamin D3, vitamin B12, and folate deficiencies were common among medical students. Exploratory analyses suggested differences in vitamin D3 and vitamin B12 concentrations across well-being groups; however, these findings did not remain significant after correction for multiple testing and should be interpreted with caution. Overall, the results indicate that lifestyle-related factors, particularly physical activity, may play a more prominent role in student well-being than serum vitamin concentrations alone. Further longitudinal studies are required to clarify these relationships.

## 1. Introduction

Due to the demanding nature of medical training, including stress, intensive studying, time pressure, limited time for preparing balanced meals, and sleep deprivation, medical students are a social group that is particularly susceptible to nutritional deficiencies and mood disturbances that may lead to serious mental health problems [1,2,3]. Global studies and literature reviews have revealed a high prevalence of reduced well-being among medical students [1,3,4]. Research has shown that one in three medical students worldwide suffers from anxiety disorders, and more than one quarter experience symptoms of depression [5,6,7]. Studies conducted at European universities have demonstrated that this problem is particularly prevalent among students in Poland, which gives cause for concern [8,9,10]. A balanced diet is a key determinant of human health and well-being [11]. In young adults, poor dietary habits are often associated with economic factors, lack of time to prepare nutritious meals, and the use of elimination diets [10,12,13,14]. Inadequate nutrition may lead to deficiencies of key nutrients, including vitamin D3, vitamin B12, and folate, which play a fundamental role in maintaining mental health [6,15]. Vitamin D3 is particularly important for central nervous system function [16,17]. It participates in genetic transcription via vitamin D receptors (VDRs). The widespread distribution and expression of VDRs across multiple organs contribute to the extensive physiological effects of vitamin D in the body. These receptors are also present throughout the brain, including in the regions that are crucial for the regulation of mood, emotions, and cognitive functions, such as the prefrontal cortex, hippocampus, hypothalamus, and substantia nigra. The mechanisms underlying the neuroprotective effects of vitamin D are complex and include the stimulation of the release of neurotransmitters such as dopamine and serotonin, which affect well-being and emotional stability [18,19,20]. Vitamin D3 also regulates neurotrophic factors and exhibits neuroimmunomodulatory and antioxidant properties, thereby supporting healthy nervous system function. In research, vitamin D3 deficiency has been increasingly associated with reduced well-being and an elevated risk of depression [21,22,23]. Relatively few food products contain vitamin D3, which is why cutaneous synthesis following exposure to ultraviolet B radiation and supplementation remain the main sources of this vitamin in the human body [16,17,24]. For this reason, vitamin D3 status is strongly influenced by geographical location and lifestyle-related factors. Cutaneous synthesis varies with latitude, season, and time spent outdoors. Consequently, individuals with limited sun exposure, including those with predominantly indoor lifestyles, may be at increased risk of vitamin D3 deficiency [25].

Due to their neurospecific functions, vitamin B12 and folate play important roles in both the central and peripheral nervous systems [17,26,27] and act as cofactors in the one-carbon metabolism pathway. Their deficiency disrupts the methylation cycle, which is believed to be involved in the pathophysiology of mental disorders [28,29]. Moreover, these vitamins are essential for the synthesis of S-adenosyl methionine (SAM), a universal methyl group donor that participates in the synthesis of neurotransmitters: serotonin, dopamine, and noradrenaline, thus potentially influencing mood and emotional regulation [18,28,30,31]. Vitamin B12 and folate are also involved in the metabolism of homocysteine to methionine. A deficiency of these nutrients increases the serum levels of homocysteine, an amino acid with well-documented neurotoxic, prothrombic, and proinflammatory effects. The homocysteine hypothesis posits that hyperhomocysteinemia is one of the risk factors for the development of mood disorders, including depression [27,32,33,34].

Current epidemiological research focuses primarily on vitamin D3 deficiencies in various age groups. Considerably less research has been done on the concentrations of B vitamins. To address this knowledge gap, the aim of the present study was to assess the prevalence of vitamin D3, vitamin B12, and folate deficiencies and to examine the relationship between the serum concentrations of these vitamins and the general well-being of medical students. This study refers to a multidimensional construct of general well-being encompassing psychological, social and emotional functioning, including mood, feeling calm and relaxed, energy levels, sleep quality, and interest in daily activities, assessed using the WHO-5 Well-Being Index.

## 2. Materials and Methods

### 2.1. Study Design

The present single-center study was conducted between May and June 2024. The participants were students enrolled in medical and paramedic programs at the University of Warmia and Mazury in Olsztyn. All participants provided informed consent to take part in the study. The study was approved by the Bioethics Committee of the Warmia and Mazury Medical Chamber in Olsztyn (decision No. 12/2024/VIII of 8 April 2024).

### 2.2. Research Methods

Venous blood samples were collected between [8:00 AM and 10:00 AM] following an overnight fast to minimize diurnal variation, once from each participant into tubes containing a clot activator. Immediately after collection, the samples were centrifuged at 4000× *g* for 10 min. Vitamin concentrations were determined in the obtained serum using an electrochemiluminescence immunoassay (ECLIA) on a fully automated Cobas e411 immunochemistry analyzer (Roche Diagnostics, Basel, Switzerland).

The participants completed author-developed online questionnaire consisting of 44 questions that collected data on demographic characteristics (age, gender, and place of residence), lifestyle factors (diet, use of stimulants, and supplementation), type and frequency of physical activity, general health and family health history and self-reported assessment of general well-being using the World Health Organization—Five Well-Being Index (WHO-5) [35]. The WHO-5 consists of five statements referring to positive mood, feeling calm and relaxed, energy levels, sleep quality, and interest in daily activities. Each item is scored on a scale of 0 (at no time) to 5 (all of the time), giving a total score of 0 to 25 points. Lower scores indicate reduced general well-being. A score of less than 13 points is suggested as an indication for further assessment for the possible presence of a mental health condition, for example, depressive disorder. The scale is intended for screening purposes, and it is not used to establish a clinical diagnosis.

### 2.3. Statistical Analysis

The obtained data were analyzed statistically using GraphPad Prism 10 software. Differences between groups were assessed with non-parametric tests, including the Kruskal–Wallis test and the Mann–Whitney U test, with nominal (unadjusted) *p*-values reported throughout. To address the issue of multiple testing, the false discovery rate was controlled using the Benjamini–Hochberg procedure for a predefined family of comparisons examining the associations between serum vitamin concentrations (vitamin D3, vitamin B12, and folate) and well-being (WHO-5 score). Both unadjusted and Benjamini–Hochberg–adjusted *p*-values for these comparisons are reported, and results after correction are interpreted as exploratory. Other analyses examining determinants of serum vitamin D3, B12 and folate concentrations were hypothesis-driven and are therefore reported using nominal *p*-values.

The WHO-5 total score is a discrete, bounded variable resulting from the sum of five ordinal items scored on a 0–5 scale, yielding integer values between 0 and 25. Preliminary inspection of the score distribution indicated deviation from normality and heteroscedasticity, limiting the suitability of classical linear regression. Therefore, a Poisson regression model was estimated as an exploratory, descriptive analysis to evaluate the association between selected key variables (gender, physical activity, and vitamin D3 supplementation) and the well-being index.

The regression model was not intended to represent a comprehensive explanatory model of well-being, but rather to assess the relative contribution of predefined variables chosen a priori based on the study objectives and existing literature. Given the limited sample size, bounded outcome range, and exploratory nature of the analysis, a parsimonious Poisson model was selected to maintain interpretability. Goodness-of-fit and overdispersion diagnostics were evaluated for each Poisson regression model.

As a sensitivity analysis, logistic regression was performed using the WHO-5 cut-off (<13 vs. ≥13) as the binary outcome.

## 3. Results

### 3.1. Characteristics of the Study Population

Participants were recruited by invitation during practical classes in diagnostics at the University of Warmia and Mazury in Olsztyn. The initial sample comprised 110 medical students and 20 paramedic students. The final study group consisted of 97 students: 80 from the medical program and 17 from the paramedic program. All eligible students present during the recruitment period were informed about the study and invited to participate. Inclusion criteria comprised: (1) current enrollment in the specified year of study, (2) age ≥ 18 years, (3) provision of informed consent, and (4) consent to biological sample collection. Exclusion criteria included refusal to participate, lack of consent for sample collection, incomplete questionnaire data, and contraindications to venipuncture, including needle-related anxiety (needle phobia). Participation was voluntary, and no incentives were offered. All participants provided informed consent prior to inclusion in the study.

The group comprised 59 women (61%) and 38 men (39%). The vast majority of the participants (92%) were aged 20–24 years. Most participants (31%) resided in cities with a population of 150,000–500,000, followed by rural areas (26%), cities with a population under 50,000 (20%), cities with a population of 50,000–150,000 (14%), and cities with a population above 500,000 (9%). A detailed description of the study population is presented in Table 1.

### 3.2. Assessment of Serum Vitamin D3, Vitamin B12, and Folate Concentrations in Medical Students

The analysis revealed that vitamin D3 concentrations were below the optimal range (30–50 ng/mL) in 78% of the students included in the study (n = 76). Within this group, 22% of the students (n = 21) had severe deficiency (<10 ng/mL), 34% (n = 33) had moderate deficiency (10–20 ng/mL), and 23% (n = 22) had suboptimal vitamin D3 concentrations (20–30 ng/mL). Optimal vitamin D3 levels were observed in only 9% of the participants (n = 9) (Table 2).

Folate deficiency was observed in 19% of the students (n = 19) (<3.89 ng/mL). Optimal levels were found in 59% of the participants (n = 57), while 21% of the students had high folate concentrations (>26.8 ng/mL) (Table 2).

Vitamin B12 deficiency was the least common and was observed in only 8% of the participants (<191 pg/mL). Optimal levels were found in 55% of the students (n = 53), while 37% had high vitamin B12 concentrations (n = 36) (>771 pg/mL) (Table 2)

### 3.3. Relationships Between Vitamin D3, Vitamin B12, and Folate Concentrations and General Well-Being in Medical Students

**Figure 1:**
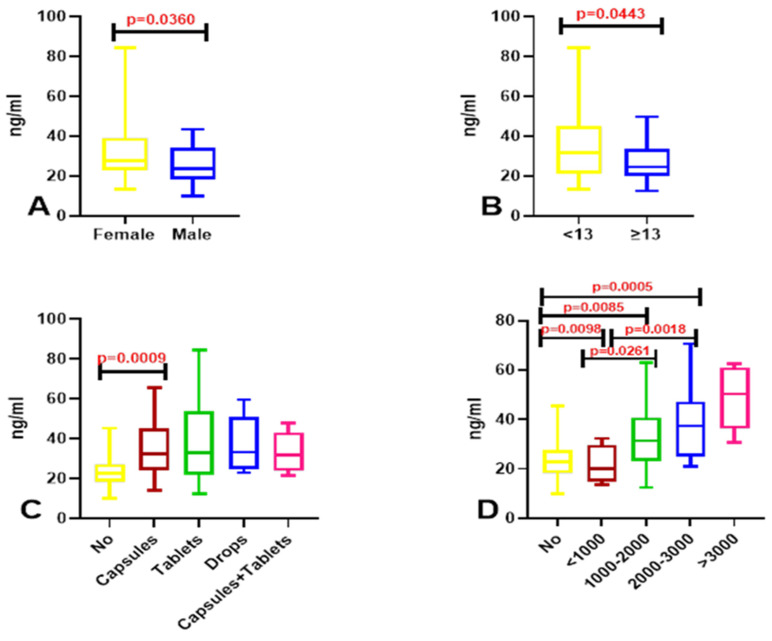
Exploratory cross-sectional analysis of serum vitamin D3 concentrations among medical students. Associations are shown in relation to gender (**A**), general well-being (WHO-5 score) (**B**), type of supplementation (**C**), and vitamin D3 intake (IU) (**D**). Data are presented as box-and-whisker plots (median, interquartile range, and min.–max. values). Statistical comparisons were performed using Mann–Whitney U tests (**A**,**B**) and Kruskal–Wallis tests with Dunn’s post hoc analysis (**C**,**D**); red *p*-values denote nominal significance (*p* < 0.05). These results represent exploratory associations within a specific cross-sectional cohort; given the sample size and potential for confounding, *p*-values should be interpreted as hypothesis-generating rather than confirmatory of a causal link.

**Figure 2:**
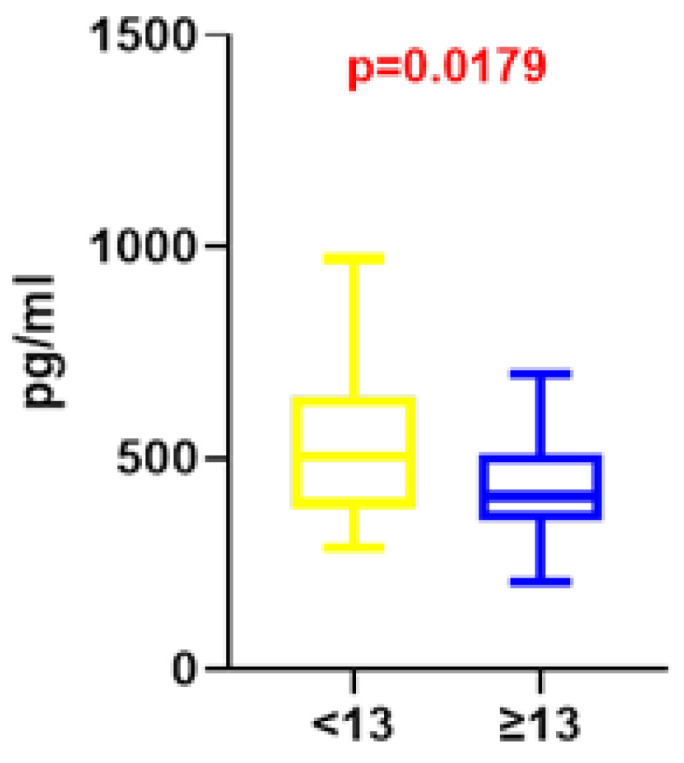
Exploratory analysis of serum vitamin B12 concentrations in relation to subjective well-being (WHO-5 score) in medical students. Data are presented as box-and-whisker plots (median, interquartile range, and min.–max. values). Statistical differences were assessed using the Mann–Whitney U test; red *p*-values denote nominal significance (*p* < 0.05). These results represent exploratory associations in a cross-sectional sample; given the sample size and the potential for confounding by unmeasured lifestyle or academic factors, these findings should be interpreted as hypothesis-generating.

In unadjusted analyses, the analysis revealed lower vitamin D3 concentrations in the group of students reporting better well-being (≥13 points on the WHO-5 scale) compared with students reporting poorer well-being (<13 points on the WHO-5 scale) (nominal *p* = 0.0443) (Figure 1B). Participants in the <13 group had a higher median vitamin D level (31.72 ng/mL, IQR: 21.33–44.98) compared with those in the ≥13 group (24.55 ng/mL, IQR: 20.20–33.46). The median difference between groups was 7.17 ng/mL, with a Hodges–Lehmann estimate of 4.84 ng/mL. The observed difference represents a small-to-moderate between-group effect. Similarly, vitamin B12 concentrations were lower in students with higher scores on the WHO-5 scale (≥13 points) than in those with lower well-being scores (<13 points) (nominal *p* = 0.0179) (Figure 2). Participants in the <13 group had a higher median vitamin B12 level (502.2 pg/mL, IQR: 382.1–645.4) compared with those in the ≥13 group (408.6 pg/mL, IQR: 350.1–509.5). The median difference between groups was 93.6 pg/mL, with a Hodges–Lehmann estimate of 62.7 pg/mL, corresponding to a moderate between-group difference. Folate concentrations were also lower in students with higher well-being scores; however, the difference was not statistically significant (nominal *p* > 0.05). The median folate level was 5.97 ng/mL (IQR: 4.33–7.88) in the <13 group and 5.42 ng/mL (IQR: 4.38–6.98) in the ≥13 group. The median difference was 0.55 ng/mL (Hodges–Lehmann estimate: 0.57 ng/mL), indicating a negligible between-group effect. After Benjamini–Hochberg correction applied to the three predefined comparisons between serum vitamin concentrations and well-being, none of the associations remained statistically significant (Supplementary Table S1). Therefore, the observed differences should be interpreted as exploratory and hypothesis-generating rather than confirmatory.

### 3.4. Assessment of Vitamin D3 Concentrations by Gender, Type of Supplementation, and Daily Vitamin D3 Intake (IU) in Medical Students

Vitamin D3 concentrations in our study group were significantly higher in women than in men (*p* = 0.0360) (Figure 1A). Significantly higher vitamin D3 levels were also observed in students supplementing vitamin D3 in capsule form compared with those not using any vitamin D3 supplements (*p* = 0.0009). Vitamin D3 concentrations were also significantly higher in students taking 2000–3000 IU than in non-supplementing individuals (*p* = 0.0005), as well as in those taking 1000–2000 IU compared with non-supplementing participants (*p* = 0.0085) and those taking less than 1000 IU compared with non-supplementing individuals (*p* = 0.0098). Vitamin D3 concentrations were significantly lower in students taking less than 1000 IU compared with those supplementing 1000–2000 IU (*p* = 0.0261) and 2000–3000 IU (*p* = 0.018) (Figure 1C,D)

### 3.5. Assessment of Folate Concentrations in Relation to BMI, Sleep Duration, and Diet in Medical Students

**Figure 3:**
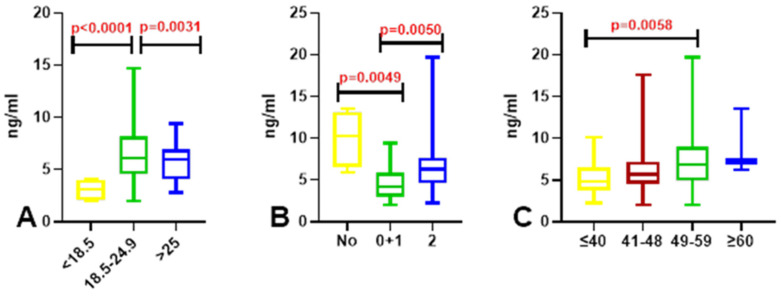
Exploratory cross-sectional comparison of serum folate concentrations in medical students. Differences are shown in relation to BMI (**A**), diet (**B**), and sleep duration (**C**). Data are presented as box-and-whisker plots (median, interquartile range, and min.–max. values). Statistical differences were assessed using the Kruskal–Wallis test; red *p*-values denote nominal significance (*p* < 0.05). Given the exploratory nature of this small-sample study and the potential for unmeasured confounding factors (e.g., lifestyle or socioeconomic variables), these *p*-values indicate trends for further investigation rather than confirmed causal relationships.

Folate concentrations were significantly lower in students from the study group with low BMI (<18.5) than in those with normal BMI (18.5–24.9) (*p* = 0.0001). Significantly higher folate concentrations were observed in students with normal BMI than in those with high BMI (>25) (*p* = 0.0031) (Figure 3A). Significantly higher folate concentrations were found in students who reported consuming fast food occasionally compared with individuals who reported consuming fast food once or several times per day or several times per week (*p* = 0.0050). Students who did not report fast food consumption at all had significantly higher folate concentrations than those who reported consuming fast food once or several times per day or several times per week (*p* = 0.0049) (Figure 3B). Folate levels were significantly higher in students sleeping 49–59 h per week compared with those sleeping less than 40 h per week (*p* = 0.0058) (Figure 3C). After correction for multiple testing using the Benjamini–Hochberg procedure, the direction and magnitude of the observed associations remained unchanged, although statistical significance was attenuated.

### 3.6. Relationships Between Gender, Physical Activity Levels, Vitamin D3 Supplementation and General Well-Being in Medical Students

A Poisson regression model (log link) fitted to 96 observations was employed to identify restricted exploratory associations between gender, physical activity, and well-being (pseudo-R^2^ = 0.0697; *df* = 92). Within this exploratory framework, gender emerged as the factor most notably associated with well-being. Specifically, men were observed to have higher expected self-reported well-being scores than women (beta = 0.1774; IRR = 1.194; 95% CI: 1.064–1.339; *p* = 0.0025), which corresponds to a potential difference of approximately 19.4% when other variables are held constant. Similarly, a trend was observed for physical activity, which was associated with higher expected well-being scores compared with inactivity (beta for inactive vs. active = −0.1208; IRR for inactivity = 0.886; 95% CI: 0.788–0.996; *p =* 0.0435). This suggests that well-being scores were approximately 12.8% higher among participants declaring regular physical activity. Vitamin D3 supplementation did not show a statistically significant association with well-being in this model (beta = −0.06145; IRR = 0.940; 95% CI: 0.838–1.055; *p* = 0.296) (Table 3). It must be emphasized that given the low pseudo-R^2^ value (0.0697), the model explains only a small proportion of the variance, suggesting that these are restricted exploratory associations and that additional unmeasured psychosocial factors likely play a more substantial role. Furthermore, as the Poisson model serves only as an approximation for the WHO-5 psychometric sum and has not been formally validated for this instrument, these preliminary findings should be interpreted with caution. Model diagnostics indicated an adequate goodness-of-fit with no evidence of substantial overdispersion.

As a sensitivity analysis, logistic regression was performed using the WHO-5 cut-off (<13 vs. ≥13) as the binary outcome. Gender and physical activity showed trends in the same direction as in the exploratory Poisson regression; however, none of the predictors reached statistical significance. Vitamin D3 supplementation was not associated with the odds of lower well-being. Overall, the logistic model showed acceptable fit (Hosmer–Lemeshow *p* = 0.75) but limited explanatory power (Tjur’s R^2^ ≈ 0.06).

## 4. Discussion

This is the first study to investigate the associations between the serum concentrations of vitamin D3, vitamin B12, and folate and general well-being (WHO-5 Index) in a cohort of medical students in Poland. Previous studies have focused primarily on vitamin D3 levels alone. In addition, the present study also evaluated whether diet and lifestyle factors influence the concentrations of the analyzed vitamins. The study population exhibited deficiencies in all three vitamins. Vitamin D3 deficiency was the most prevalent, affecting approximately 78% of participants, which aligns with both domestic and international reports regarding medical student populations [36,37,38,39,40]. The present findings and the cited studies suggest that the lifestyle associated with medical studies, which is characterized by high stress levels, limited time for preparing balanced meals, and reduced sun exposure, may represent a common risk factor for vitamin D3 deficiency across student populations, regardless of geographic location. Therefore, maintaining adequate vitamin D3 levels among university students in Poland may require increased consumption of foods rich in this vitamin and supplementation based on current scientific guidelines, particularly in fall and winter, when sun exposure is reduced [41,42,43,44,45].

Analysis by gender revealed significantly higher mean vitamin D3 levels in female students compared to males (*p* = 0.0360). This is noteworthy because several population-based studies have identified female gender as a risk factor for deficiency [39,42,46,47]. Research indicates that women are generally more likely to engage in health-promoting behaviors and use dietary supplements. Thus, female medical students might use supplements more consistently than their male counterparts [16,37,40,43,48]. However, this remains a hypothesis, as questionnaire limitations preclude definitive conclusions.

In the current study, folate deficiency affected 19% of medical students of the University of Warmia and Mazury in Olsztyn. Hypothetically, this deficiency could be partially explained by an inadequate diet and lack of supplementation [49,50,51]. The observed significant association between lower folate levels and more frequent consumption of fast food (as reported by respondents) may indicate that a diet based largely on highly processed foods has a negative impact on nutritional status. This finding is consistent with previous studies suggesting that the Western diet is generally poor in natural sources of folate—fresh, green leafy vegetables, legumes, and whole grains, which are the richest sources of this vitamin [26,50]. Folate is particularly sensitive to thermal processing, including frying and baking, and losses of this vitamin during food preparation can range from 50% to as much as 90%. Dietary patterns characteristic of the Western diet are considered one of the major risk factors for folate deficiency in developed countries, especially among students, who often lack the time and financial resources to prepare fresh, nutritionally balanced meals [26,50,51,52]. Furthermore, a study conducted among Polish female university students revealed very low awareness of folic acid supplementation recommendations as well as a low proportion of women who use such supplements [50,51].

The association observed between lower folate concentrations and both underweight (BMI < 18.5) and overweight (BMI > 25) students may suggest that individuals with either very low or excessive body weight are more likely to follow restrictive or nutritionally imbalanced diets [52,53,54]. It is worth noting that both underweight and overweight are forms of malnutrition and may also be associated with vitamin deficiencies [13,22]. An analysis of the correlations between sleep duration and folate concentrations points to a complex relationship. Both very short sleep duration (less than 6 h per night) and very long sleep duration (more than 9 h per night) showed a tendency toward lower folate concentrations relative to students with optimal sleep duration (7–8 h per night). It can be assumed that this relationship is bidirectional and reflects the key role of B vitamins, including folic acid, in the functioning of the central nervous system. Folate is essential for the synthesis of neurotransmitters, including serotonin, which is a precursor of melatonin, the hormone regulating the sleep–wake cycle. As a result, folate deficiency may disrupt the production of these neurotransmitters, potentially contributing to difficulties with sleep initiation, lower sleep quality, and symptoms such as fatigue, apathy, and irritability [55,56,57]. Suboptimal sleep duration, whether too short or too long, is often considered a marker of an unhealthy lifestyle, chronic stress, and poor dietary habits. Individuals who regularly experience insufficient or excessive sleep may also be more likely to make unhealthy dietary choices, such as consuming highly processed foods, which can contribute to lower folate levels. Due to their heavy academic workload, students are particularly prone to irregular sleep patterns [58,59,60].

The prevalence of vitamin B12 deficiency was low, affecting only 8% of the students, which is consistent with expectations given the relatively large body stores of this vitamin and its presence in animal-derived foods that form part of a typical Polish diet, as well as the small proportion of vegans and vegetarians in the study population [50,60,61].

The analysis of the relationships between vitamin D3, vitamin B12, and folate concentrations and the students’ self-reported well-being produced surprising results that contrast with many literature reports. In terms of absolute values, students included in our study with lower psychological well-being (WHO-5 < 13) had a median serum vitamin D3 concentration of 31.7 ng/mL (IQR: 21.3–45.0), compared with 24.6 ng/mL (IQR: 20.2–33.5) in those with better well-being (WHO-5 ≥ 13). Although nominally significant in unadjusted analyses, the observed between-group difference of approximately 5–7 ng/mL was modest, and median values in both groups remained close to commonly accepted thresholds for vitamin D sufficiency.

This finding is surprising in light of several meta-analyses that revealed a strong association between low vitamin D concentrations and depressed mood or an increased risk of depression in young adults [11,19,20,62,63,64], which constituted one of the premises for conducting the present study. In turn, vitamin B12 and folate deficiencies contribute to hyperhomocysteinemia and impaired neurotransmitter synthesis and are well-established risk factors for mood disorders [15,20,28,30,65,66,67]. Unexpectedly, our data suggested a potential inverse pattern between general self-reported well-being and concentrations of vitamin D3 and B12 in unadjusted analyses. While biologically counter-intuitive, this likely reflects reverse causation or confounding factors rather than a negative impact of vitamins on mental health. It is possible that students with poorer well-being scores had already initiated supplementation—a common response to academic burnout or fatigue—leading to the observed higher serum levels at the time of the venipuncture. Because our study did not precisely track the duration, dosage, or indication for supplementation, we cannot confirm this trajectory. This highlights a primary limitation of the cross-sectional design: it captures a static moment in a dynamic process of health-seeking behavior. Moreover, the general well-being of medical students in our study (also observed in other studies of Polish medical students) is influenced by numerous stress-related factors associated with academic pressure, insufficient sleep, limited physical activity, and the use of stimulants [1,68,69,70]. Therefore, it is possible that these stressors exerted a stronger influence on the participants’ general self-reported well-being than vitamin deficiencies alone. This unexpected finding warrants further analysis of the potential mechanisms underpinning the observations made in the study population.

A Poisson regression model was applied to assess the potential impact of gender, physical activity, and vitamin D3 supplementation on the well-being of medical students in our study group. The analysis revealed that well-being was associated with gender and physical activity but not with vitamin D3 supplementation. The model also demonstrated that male students had higher expected well-being scores than female students (IRR = 1.194; 95% CI: 1.064–1.339; *p* = 0.0025). This result may be related to gender differences in psychological well-being, stress responses, or perceived workload burden that are well documented in the literature. Many population studies have shown that women report symptoms of low mood and higher stress levels more often than men, which may explain the lower well-being scores of women in the current study. However, these mechanisms were not directly assessed in the present study, and any such explanations remain hypothetical. To address potential limitations of using Poisson regression for a bounded psychometric score, a sensitivity analysis was conducted using logistic regression based on the WHO-5 cut-off (<13 vs. ≥13). This analysis yielded a similar overall pattern of associations, with no statistically significant effects observed, supporting cautious interpretation of the exploratory multivariable findings.

Physical activity was also a significant factor that was associated with approximately 13% higher well-being scores compared with physically inactive students in our study group (IRR = 1.128; 95% CI: 1.004–1.270; *p* = 0.0435). Similar observations have been made in numerous studies, which indicate that regular physical activity supports emotional regulation, reduces stress symptoms, and increases energy levels. This finding underscores the importance of health-promoting behaviors as the key factors of psychological well-being.

Unlike physical activity, vitamin D3 supplementation in our study group was not significantly associated with well-being (IRR = 0.940; *p* = 0.296). It should be noted that the effects of supplementation may be influenced by baseline serum vitamin D3 levels, dosage, and the regularity of supplementation. The lack of statistical significance in the model does not necessarily exclude a potential effect but rather points to the need for more detailed measurements or a larger population sample. Moreover, well-being is a multifactorial outcome that is influenced by many biological and psychosocial variables, which may attenuate the observable effects of supplementation. In addition, the overall fit of the model (pseudo-R^2^ = 0.0697) indicates that the analyzed variables explain approximately 7% of the variance in well-being. This implies that well-being may be more influenced by factors that were not included in the model, such as stress, sleep quality, social relationships, mental and physical health, dietary habits, BMI, use of stimulants and socioeconomic conditions. Their omission reflects both the exploratory nature of this analysis and the limited sample size, which constrained the inclusion of a larger number of predictors without risking model overfitting. Consequently, residual confounding cannot be excluded. Therefore, the present findings may be interpreted as meaningful, but only partially explain the observed variation in well-being. Variables such as sleep quality, diet, BMI or stimulant use should be incorporated into multivariable models in future studies with larger samples.

The interdependence of behavioral and environmental variables that were not included in the model could be a limitation of the present study. For example, physically active individuals may also differ in social functioning, dietary habits, or perceived self-efficacy that could influence well-being. The choice of Poisson regression for modeling WHO-5 scores represents a methodological compromise. While the outcome variable is discrete and bounded, it does not strictly follow a Poisson distribution, and the WHO-5 was not originally designed as a count measure. Consequently, the applied model should be viewed as an exploratory tool for assessing relative associations, rather than as a definitive psychometric model.

In summary, the present results suggest that physical activity and gender differences may be associated with self-reported well-being in the studied sample, whereas no significant association with vitamin D3 supplementation was observed. In contrast to the strong emphasis placed on vitamin D3 in the existing literature on mood regulation, our findings indicate that lifestyle-related factors, particularly physical activity, may play a more prominent role in well-being among medical and paramedic students.

Several methodological limitations should be acknowledged. The study population was highly age-homogeneous, with most participants aged between 20 and 24 years, which limited the assessment of age-related effects. Moreover, the inclusion of both medical and paramedic students without separate subgroup analyses may have obscured potential program-specific differences in lifestyle and stress exposure. The WHO-5 is a self-reported screening instrument rather than a diagnostic tool, and the cross-sectional design precludes causal inference. The relatively small sample size and single-center design further limit statistical power and generalizability. Importantly, the large number of statistical comparisons performed increases the risk of type I error; therefore, marginal *p*-values should be interpreted with caution and regarded as hypothesis-generating rather than definitive. The restricted scope of the multivariable regression analysis, which did not simultaneously include several relevant lifestyle and psychosocial variables, further underscores the exploratory nature of the findings.

Finally, the timing of data collection represents an additional limitation. Serum vitamin D3 concentrations were measured in late spring (May–June), when endogenous synthesis begins to increase at northern latitudes. Consequently, the prevalence of vitamin D insufficiency observed in this study may underestimate levels during the winter months. Future studies with larger samples, longitudinal designs, and more comprehensive multivariable models are needed to clarify the complex relationships between vitamin status, lifestyle factors, and well-being in student populations.

## 5. Conclusions

Vitamin D3, vitamin B12, and folate deficiencies were common among medical students. Exploratory analyses suggested differences in vitamin D3 and vitamin B12 concentrations across well-being groups; however, these findings did not remain significant after correction for multiple testing and should be interpreted with caution. Overall, the results indicate that lifestyle-related factors, particularly physical activity, may play a more prominent role in student well-being than serum vitamin concentrations alone. Further longitudinal studies are required to clarify these relationships.

## Figures and Tables

**Table 1:** Study population characteristics.

	n %	Vitamin B12 Concentrations			Vitamin D3 Concentrations						Folate Concentrations		
Number of Participants	97 (100%)	Deficiency	Optimal	High	Severe Deficiency	Considerable Deficiency	Moderate Deficiency (Suboptimal)	Optimal	High	Toxic	Deficiency	Optimal	High
Age													
<20	1 (1%)	0 (0%)	1 (1%)	0 (0%)	1 (1%)	0 (0%)	0 (0%)	0 (0%)	0 (0%)	0 (0%)	0 (0%)	1 (1%)	0 (0%)
20–24	89 (92%)	8 (8%)	49 (51%)	32 (33%)	20 (21%)	30 (31%)	21 (22%)	9 (9%)	3 (3%)	6 (6%)	19 (20%)	54 (56%)	16 (16%)
25–29	3 (3%)	0 (0%)	2 (2%)	1 (1%)	0 (0%)	2 (2%)	1 (1%)	0 (0%)	0 (0%)	0 (0%)	0 (0%)	1 (1%)	2 (2%)
≥30	4 (4%)	0 (0%)	1 (1%)	3 (3%)	0 (0%)	1 (1%)	0 (0%)	0 (0%)	3 (3%)	0 (0%)	0 (0%)	1 (1%)	3 (3%)
Gender													
Female	59 (61%)	7 (7%)	27 (28%)	25 (26%)	9 (9%)	22 (23%)	14 (14%)	7 (7%)	2 (2%)	5 (5%)	12 (12%)	32 (33%)	15 (15%)
Male	38 (39%)	1 (1%)	26 (27%)	11 (11%)	12 (12%)	11 (11%)	8 (8%)	2 (2%)	4 (4%)	1 (1%)	7 (7%)	25 (26%)	6 (6%)
Place of residence													
City with a population of 150,000 to 500,000	30 (31%)	1 (1%)	17 (18%)	12 (12%)	7 (7%)	6 (6%)	9 (9%)	3 (3%)	3 (3%)	2 (2%)	3 (3%)	20 (21%)	7 (7%)
City with a population up to 50,000	19 (20%)	3 (3%)	8 (8%)	8 (8%)	4 (4%)	10 (10%)	2 (2%)	1 (1%)	1 (1%)	1 (1%)	7 (7%)	10 (10%)	2 (2%)
Rural area	25 (26%)	2 (2%)	17 (18%)	6 (6%)	5 (5%)	11 (11%)	5 (5%)	3 (3%)	1 (1%)	0 (0%)	3 (3%)	13 (13%)	9 (9%)
City with a population of 50,000 to 150,000	14 (14%)	1 (1%)	5 (5%)	8 (8%)	3 (3%)	4 (4%)	4 (4%)	0 (0%)	1 (1%)	2 (2%)	2 (2%)	10 (10%)	2 (2%)
City with a population above 500,000	9 (9%)	1 (1%)	6 (6%)	2 (2%)	2 (2%)	2 (2%)	2 (2%)	2 (2%)	0 (0%)	1 (1%)	4 (4%)	4 (4%)	1 (1%)
Well-being (WHO-5 score)													
<13	49 (51%)	4 (4%)	22 (23%)	23 (24%)	10 (10%)	13 (13%)	12 (12%)	4 (4%)	4 (4%)	6 (6%)	9 (9%)	27 (28%)	13 (13%)
≥13	48 (49%)	4 (4%)	31 (32%)	13 (13%)	11 (11%)	20 (21%)	10 (10%)	5 (5%)	2 (2%)	0 (0%)	10 (10%)	30 (31%)	8 (8%)
BMI													
<18.5	6 (6%)	2 (2%)	3 (3%)	1 (1%)	1 (1%)	5 (5%)	0 (0%)	0 (0%)	0 (0%)	0 (0%)	4 (4%)	1 (1%)	1 (1%)
18.5–24.9	68 (70%)	5 (5%)	33 (34%)	30 (31%)	12 (12%)	22 (23%)	18 (19%)	7 (7%)	4 (4%)	5 (5%)	9 (9%)	40 (41%)	19 (20%)
25–29.9	21 (22%)	1 (1%)	17 (18%)	3 (3%)	7 (7%)	6 (6%)	3 (3%)	2 (2%)	2 (2%)	1 (1%)	6 (6%)	14 (14%)	1 (1%)
30–34.9	2 (2%)	0 (0%)	0 (0%)	2 (2%)	1 (1%)	0 (0%)	1 (1%)	0 (0%)	0 (0%)	0 (0%)	0 (0%)	2 (2%)	0 (0%)
35–39.9	0 (0%)	0 (0%)	0 (0%)	0 (0%)	0 (0%)	0 (0%)	0 (0%)	0 (0%)	0 (0%)	0 (0%)	0 (0%)	0 (0%)	0 (0%)
≥40	0 (0%)	0 (0%)	0 (0%)	0 (0%)	0 (0%)	0 (0%)	0 (0%)	0 (0%)	0 (0%)	0 (0%)	0 (0%)	0 (0%)	0 (0%)
Frequency of physical activity													
More than 4 times per week	5 (5%)	0 (0%)	2 (2%)	3 (3%)	0 (0%)	2 (2%)	2 (2%)	1 (1%)	0 (0%)	0 (0%)	1 (1%)	4 (4%)	0 (0%)
None	27 (28%)	5 (5%)	9 (9%)	13 (13%)	7 (7%)	10 (10%)	5 (5%)	1 (1%)	1 (1%)	3 (3%)	8 (8%)	15 (15%)	4 (4%)
Once a week	36 (37%)	2 (2%)	24 (25%)	10 (10%)	9 (9%)	9 (9%)	8 (8%)	6 (6%)	2 (2%)	2 (2%)	4 (4%)	23 (24%)	9 (9%)
Twice a week	18 (19%)	1 (1%)	10 (10%)	7 (7%)	3 (3%)	9 (9%)	3 (3%)	1 (1%)	1 (1%)	1 (1%)	4 (4%)	10 (10%)	4 (4%)
Three times per week	11 (11%)	0 (0%)	8 (8%)	3 (3%)	2 (2%)	3 (3%)	4 (4%)	0 (0%)	2 (2%)	0 (0%)	2 (2%)	5 (5%)	4 (4%)
Supplementation													
None	-	8 (8%)	41 (42%)	31 (32%)	17 (18%)	16 (16%)	6 (6%)	2 (2%)	0 (0%)	0 (0%)	15 (15%)	54 (56%)	15 (15%)
On a regular basis	-	0 (0%)	0 (0%)	1 (1%)	1 (1%)	0 (0%)	1 (1%)	3 (3%)	3 (3%)	2 (2%)	0 (0%)	0 (0%)	1 (1%)
Only in fall and winter	-	0 (0%)	0 (0%)	0 (0%)	0 (0%)	4 (4%)	6 (6%)	1 (1%)	1 (1%)	2 (2%)	0 (0%)	0 (0%)	0 (0%)
Not on a regular basis	-	0 (0%)	12 (12%)	4 (4%)	3 (3%)	13 (13%)	9 (9%)	3 (3%)	2 (2%)	2 (2%)	4 (4%)	3 (3%)	5 (5%)

**Table 2:** Serum vitamin D3, B12, and folate concentrations in medical students.

Vitamin	Median	IQR (25th–75th)	(Min–Max)	Reference Value *
D3 (ng/mL)	31.2	21.71–36.84	9.9–84.5	30–50
B12 (pg/mL)	477.8	351.4–569.2	122–1372	191–771
Folate (ng/mL)	6.7	4.405–7.285	3.89–20.0	3.89–26.8

* vitamin D3 was reported the optimal concentration (low level < 20 ng/mL; suboptimal 20–30 ng/mL; high 50–100 ng/mL; toxic > 100 ng/mL).

**Table 3:** Parameters of the Poisson regression model for the “well-being” dependent variable.

Predictor	Coefficient β (SE)	IRR (95% CI)	Z	*p*-Value
Constant (Intercept)	2.566 (0.047)	(2.472–2.657)	54.53	<0.0001
Gender (male vs. female)	0.177 (0.059)	1.194 (1.064–1.339)	3.02	0.0025
Physical activity (yes vs. no)	0.121 (0.060)	1.128 (1.004–1.270)	2.02	0.0435
Vitamin D3 supplementation (yes vs. no)	−0.061 (0.059)	0.940 (0.838–1.055)	1.05	0.2960

## Data Availability

Data is contained within the article.

## Supplementary Materials

The following supporting information can be downloaded at: https://www.mdpi.com/article/10.3390/nu18101559/s1, Table S1: Comparisons between serum vitamin concentrations and well-being.
