# Supplementary material for: Associations Between Concentrations of Vitamin D3, Vitamin B12, and Folate and the Well-Being of Medical Students
Source: Nutrients. 2026 May 14;18(10):1559. doi: 10.3390/nu18101559 (PMC13209173; doi:10.3390/nu18101559)
Supplement: Supplementary file 1 [file nutrients-18-01559-s001.zip › nutrients-4253957-supplementary.pdf]

Table S1. Comparisons between serum vitamin concentrations and well-being.

Comparison	p (unadjusted)	p (BH-adjusted)
D3 vs WHO-5	0.0443	0.0665
B12 vs WHO-5	0.0179	0.0537
Folate vs WHO-5	0.2895	0.2895
